# *Notes from the Field*: COVID-19 Case Investigation and Contact Tracing Program — Spirit Lake Tribe, North Dakota, September–November 2020

**DOI:** 10.15585/mmwr.mm7014a4

**Published:** 2021-04-09

**Authors:** James Matthias, Tracy Charboneau, Cheri Schaffer, Jennifer Rusten, Sharon Whitmer, Joseph de la Paz, Janet Dykstra, Ishani Pathmanathan, Daniel Stowell

**Affiliations:** ^1^CDC COVID-19 Response Team; ^2^Spirit Lake Tribal Health, Spirit Lake Tribe, North Dakota; ^3^Spirit Lake Health Center, Spirit Lake Tribe, North Dakota.

In late September 2020, the incidence of confirmed COVID-19* in North Dakota began increasing rapidly, from approximately 300 new cases per day to approximately 2,260 cases on November 13, 2020 (*1*). On October 20, the North Dakota Department of Health reported that contact tracing notification efforts were delayed. Because of the delay, COVID-19 patients were asked to notify their own contacts about potential exposure and encourage them to seek testing for SARS-CoV-2, the virus that causes COVID-19 (*2*). The Spirit Lake sovereign nation in east central North Dakota is home to approximately 7,500 members of the Spirit Lake Tribe. In response to increasing incidence of COVID-19 on the Spirit Lake Reservation, CDC assisted the Spirit Lake Tribe in building a tribally managed program for comprehensive COVID-19 case investigations, case notification, contact tracing, contact testing, and contact management to ensure timely implementation of these critical epidemic control measures.

Through the Spirit Lake Tribe case investigation and contact tracing program, the tribe’s COVID-19 Incident Command System staff members conducted case investigations and contact tracing, provided COVID-19 education, followed up with patients regularly by telephone, and monitored daily symptoms of close contacts. Members of the Spirit Lake community served as contact tracers. Symptom monitoring was facilitated through CDC’s Text Illness Monitoring system (version TIM^2^), using a free, two-way text-messaging platform to query enrolled contacts about daily COVID-19 symptoms. The system also alerted Spirit Lake Tribal Health authorities when participants reported symptoms or did not to respond (*3*). This report describes case investigation and contact tracing for the Spirit Lake Tribe during September 29, 2020 (when the case and contact tracing launched) through November 20, 2020 (when the CDC field response ended) and lessons learned from program implementation. This activity was reviewed by CDC and was conducted consistent with applicable federal law and CDC policy.^†^

During September 29–November 20, data were retrieved by COVID-19 response team members from Spirit Lake Tribal Health’s COVID-19 case and contact database, including patient demographics; links between COVID-19 patients and close contacts; test results; and symptom onset, isolation, and quarantine dates. Symptom data from Spirit Lake’s TIM^2^ monitoring system were analyzed to assess COVID-19 symptoms reported by close contacts of COVID-19 patients during October 22, (when TIM^2^ use began for contact management) through November 30 (when the last close contact unit^§^ enrolled by November 20 completed quarantine).

During September 29–November 20, a total of 317 persons with confirmed COVID-19 and 667 close contacts among the Spirit Lake Tribe were reported; 129 (19.3%) of these close contacts received a subsequent COVID-19 diagnosis (Table). The average interval between specimen collection to receipt of a positive SARS-CoV-2 test result was 2.25 days (median = 3 days, range = 0–9 days). Overall, 254 (80.1%) of 317 patients with confirmed COVID-19 and 420 (78.1%) of 538 close contacts who did not receive a COVID-19 diagnosis were contacted by program staff members and instructed to isolate or quarantine within 24 hours of receipt of test results or identification of cases.^¶^ The proportion of confirmed new COVID-19 cases arising from known contacts was 41% (weekly range = 24%–59%).

**TABLE:** Number of patients with confirmed COVID-19 and close contacts who did or did not receive a COVID-19 diagnosis ― Spirit Lake Tribe, North Dakota, September 29–November 20, 2020*

Dates^†^	No. of patients with confirmed COVID-19 (% of cases from close contacts)^§^	Close contacts		
		No. who received a COVID-19 diagnosis (%)^§^	No. who did not receive a COVID-19 diagnosis^¶^	Total no.
September 29–October 2**	35 (20)	7 (10)	61	**68**
October 3–9	39 (59)	23 (23)	75	**98**
October 10–16	39 (41)	16 (18)	74	**90**
October 17–23**^††^**	40 (50)	20 (25)	60	**80**
October 24–30	40 (38)	15 (15)	88	**103**
October 31–November 6	42 (36)	15 (26)	43	**58**
November 7–13	37 (24)	9 (9)	88	**97**
November 14–20	45 (53)	24 (33)	49	**73**
**Total**	**317 (41)**	**129 (19)**	**538**	**667**

* These numbers might not include all patients or close contacts associated with the Spirit Lake Tribe because of limited sharing of health information among overlapping state, local, and tribal jurisdictions.

**^†^** The date used for confirmed COVID-19 patients was the date of receipt of a positive SARS-CoV-2 test result; for close contacts the date used was the date of identification as a close contact by a confirmed COVID-19 patient.

^§^ Close contacts who received a COVID-19 diagnosis were included in the total number of patients with confirmed COVID-19 on the date that they received their positive test result. The percentage of cases among close contacts was calculated as the number of close contacts who received a COVID-19 diagnosis divided by the number of confirmed COVID-19 cases.

^¶^ Included close contacts who received a negative SARS-CoV-2 test result, those who did not receive testing after exposure, and SARS-CoV-2 infections not reported to Spirit Lake Tribal Health and Spirit Lake Health Center.

** This week only contains 4 days to align with the assessed period (September 29–November).

**^††^** During this and all subsequent weeks, close contacts of confirmed COVID-19 patients were given the option to enroll in CDC’s Text Illness Monitoring system (version TIM^2^) for daily symptom monitoring.

During October 22–November 30, a total of 44 close contact units were enrolled in TIM^2^, which logged 366 responses during 524 quarantine days (70% daily response rate). Among these 44 enrolled close contact units, 17 (39%) reported one or more persons with symptoms, 16 (94%) of whom were contacted within 24 hours and instructed to quarantine to prevent further transmission. A total of 20 persons from eight close contact units received a COVID-19 diagnosis. During the assessment period, the incidence of COVID-19 in the Spirit Lake Tribe plateaued at approximately 520–600 cases per 100,000 persons per week; during the same period, a 1.5-fold increase in incidence occurred in North Dakota, from 455 to 1,137 cases per 100,000 per week (*1*,*4*).

Implementation of a COVID-19 case investigation and contact tracing program for the Spirit Lake Tribe highlighted several important lessons. First, the program required daily, continuous staffing to effect timely COVID-19 mitigation. Second, obtaining information from and maintaining contact with COVID-19 patients and their close contacts was challenging. Using Spirit Lake community members as investigators and contact tracers aided in outreach because of their knowledge of alternate methods to reach patients or contacts (in-home or family contacts) when locating information was incomplete. These community members also helped to improve response rates about COVID-19 exposures because they were trusted by the community and were able to provide culturally appropriate advice about the need to isolate or quarantine. Third, shared rooms and living spaces among multigenerational families or within whole households with cases or exposures in this community often hindered within-home quarantine and isolation and adherence to these measures. To address this challenge, program staff members distributed critical supplies (e.g., groceries, over-the-counter medications, thermometers, personal protective equipment, and cleaning supplies) and health literacy information (about daily temperature logs, isolation and quarantine procedures, and mask use) to each household. Finally, approximately 100 (10%) persons identified through the case investigation and contact tracing program experienced homelessness or unstable housing during this period, necessitating the provision of temporary shelter and meals at a motel for these persons during isolation and quarantine.

Despite these challenges, this tribally managed COVID-19 case investigation and contact tracing program effectively reached Spirit Lake tribal members to provide isolation, quarantine, symptom monitoring, and support services and contributed to timely case and contact management. This program might help guide similar programs in other tribes and the public health community.

## Footnotes

* The COVID-19 case definition followed CDC guidelines from September 2020. https://wwwn.cdc.gov/nndss/conditions/coronavirus-disease-2019-covid-19/case-definition/2020/08/05/

† 45 C.F.R. part 46.102(l)(2), 21 C.F.R. part 56; 42 U.S.C. Sect. 241(d); 5 U.S.C. Sect. 552a; 44 U.S.C. Sect. 3501 et seq.

§ Individual persons or family units sharing a phone number.

¶ Patients were instructed to isolate in accordance with CDC guidance at the time (https://www.cdc.gov/coronavirus/2019-ncov/hcp/duration-isolation.html), and close contacts were instructed to quarantine in accordance with CDC guidance at the time, which was 14 days after last exposure to a COVID-19 patient.
